# Seasonal Effect on Feed Intake and Methane Emissions of Cow–Calf Systems on Native Grassland with Variable Herbage Allowance

**DOI:** 10.3390/ani11030882

**Published:** 2021-03-19

**Authors:** M. Soledad Orcasberro, Cecilia Loza, José Gere, Pablo Soca, Valentín Picasso, Laura Astigarraga

**Affiliations:** 1 Departamento de Producción Animal y Pasturas, Facultad de Agronomía, Universidad de la Republica, Montevideo 12900, Uruguay; cloza@fagro.edu.uy (C.L.); psoca@fagro.edu.uy (P.S.); picassorisso@wisc.edu (V.P.); astigarr@fagro.edu.uy (L.A.); 2 Unidad de Investigación y Desarrollo de las Ingenierías (UTNBA), Consejo Nacional de Investigaciones Científicas y Técnicas (CONICET), Ciudad Autónoma de Buenos Aires C1179AAQ, Argentina; jgere@frba.utn.edu.ar; 3 Department of Agronomy, University of Wisconsin, Madison, WI 53706, USA

**Keywords:** greenhouse gases, methane yield, beef cows

## Abstract

**Simple Summary:**

Improving grazing management provides the opportunity of limiting methane emissions from beef cattle systems, and consequently offers economic along with environmental benefits. The aim of this study was to measure methane emissions and herbage intake, in order to estimate the methane yield from beef cows grazing on native grasslands at different herbage allowances. The trial, that it is part of a long-term experiment, consisted in two treatments of herbage allowance, with forty pregnant heifers. Methane emissions and intake were estimated for three 17-day periods during autumn, winter and spring. Methane emissions and organic matter intake did not differ between herbage allowance treatments, which resulted in similar methane yield. However, all variables were significantly affected by the period, with a marked increase in spring, except for methane yield expressed as a proportion of Gross Energy intake. Results show that methane emissions and intake were significantly affected by the season of the year, but not by the level of herbage allowance used in this study. These are the first data obtained on methane emissions in pregnant heifers in native grassland for Uruguay.

**Abstract:**

The aim of this study was to measure methane emissions (CH_4_) and herbage intake, and, on the basis of these results, obtain the methane yield (MY, methane yield as g CH_4_/kg dry matter intake (DMI) and Ym, methane yield as a percentage of Gross Energy intake), from beef cows grazing on native grasslands. We used forty pregnant heifers, with two treatments of herbage allowance (HA) adjusted seasonally (8 and 5 kg dry matter (DM)/kg cattle live weight (LW), on average), during autumn, winter and spring. Methane emissions (207 g CH_4_/d), organic matter intake (OMI, 7.7 kg organic matter (OM)/d), MY (23.6 g CH_4_/kg DMI) and Ym (7.4%), were similar between treatments. On the other hand, all variables had a marked increase in spring (10.8 kg OM/d and 312 g CH_4_/d), except for Ym. The methane emission factor from Intergovernmental Panel on Climate Change (IPCC) Tier 2 estimated with these results was 78 kg CH_4_/head/year. The results show that methane emissions and intake were influenced by the season, but not by the HA analyzed in this study. This information for cow–calf systems in native grasslands in Uruguay can be used in National greenhouse gases (GHG) inventories, representing a relevant contribution to global GHG inventories.

## 1. Introduction

Extensive grazing systems used by ruminants have been the focus of multiple criticisms due to their attribution to high greenhouse gas emissions (GHG). Livestock production is growing worldwide because of the increased demand for animal proteins. Beef cattle production has increased in the last three decades almost 40% worldwide, with the Americas being one of the regions that led this development [1]. However, beef produced from native grassland ecosystems provides multiple benefits such as biodiversity conservation, low energy and inputs consumption, carbon sequestration in soils, improved animal welfare, and rural development [2,3].

Uruguayan grasslands are located in the Campos region [4,5,6,7], and its export competitiveness is based in beef production at grazing all year round. Though, enteric methane emissions from livestock sector, mainly the cow–calf phase, explain about 75% of the 2010 National GHG Inventory [8]. Cow–calf systems in the Campos region are characterized by low pregnancy rates [9,10], and low calf weaning weight [11].

After the CoP21 meeting in Paris, there was a multinational agreement to act against climate change and that includes interventions in the livestock sector, by reducing methane emissions per unit of product (carbon footprint), in association with the promotion of sustainable grazing practices, so that the associated benefits of pastoralism are not sacrificed [3]. In this sense, using herbage mass efficiently by managing allowance per animal, is a key mitigation option in beef cow–calf grazing systems that can increase beef productivity and reduce carbon footprint [1].

Management practices adopted by some farmers such as improving grazing management by adjusting stocking rate seasonally [12], have the potential to mitigate GHG emissions due to the direct effect on improvement of animal performance and thus, resulting in lower GHG emissions per unit of output [13,14,15,16]. Recent experimental evidence in Uruguay confirmed that optimizing herbage management by controlling seasonal herbage allowance (HA, defined as kilograms of herbage dry matter (DM) per kilogram of animal live weight (LW)) [4,17] increased pasture productivity, reproductive performance and beef productivity in cow–calf systems [18,19,20,21,22,23]. This management generates fluctuations in the production and accumulation of herbage that increases biomass in spring and summer. The concept behind this strategy is to maintain greater herbage mass during the grass growing season (spring and summer) to be consumed during the period when herbage growth drops or becomes zero [20,21,22].

However, the impact of managing seasonal HA on methane emissions has not been quantified yet even though methane (CH_4_) is the main GHG in cow calf systems [14]. Therefore, the aim of this study was to measure methane emissions related to enteric fermentation and herbage intake from beef cows grazing on native grassland at two HA (high, HHA and low, LHA) with seasonal variations, in order to estimate the methane yield of this system. Our hypotheses were: (1) herbage mass and energy intake would be higher in the high vs. low HA treatment, (2) methane yield would be lower in the high vs. low HA treatments.

## 2. Materials and Methods

### 2.1. Experimental Treatments and Design

The experiment was conducted at the Bernardo Rosengurtt Research Station, Universidad de la República, Uruguay (32°22′ S, 54°26′ W), from autumn to spring 2015. The region (north-east of Uruguay) has a subtropical climate, very warm in summer but with frosts in winter. The average of the coldest month (July) is 11.7 °C, and the average of the hottest month (January) is 23.8 °C [24]. Average annual precipitation ranges between 1300 and 1400 mm, with a low variation between months. The soil moisture balance shows periods of excess (precipitation higher than evapotranspiration) in autumn and winter and deficits in summer [25].

The experiment was on native grasslands that have been extensively studied as a long-term research site [19]. Dominant warm-season grasses (C4) are *Andropogon ternatus*, *Axonopus affinis*, *Bothriochloa laguroides*, *Mnesithea selloana*, *Paspalum dilatatum*, *Paspalum notatum*, *Paspalum plicatulum*, *Schizachyrium microstachyum*, *Sporobolus indicus*, *Steinchisma hians*. They contributed to 75%, 54%, 58% of total grasses in autumn, winter and spring, respectively. Cool-season grasses (C3) are *Nassella charruana*, *Nassella mucronata*, *Piptochaetium stipoides*. They represent 9%, 9%, 8% of total grasses in autumn, winter and spring, respectively. These grasses (C3 and C4) provide the majority of livestock forage [26].

Treatments consisted in two HA under continuous grazing, with seasonal variations. Herbage allowance, defined as kilograms of herbage dry matter (DM) per kilogram of animal live weight (LW), was established according to the methodology of Sollenberger et al. [17]. High herbage allowance (HHA) was 8 kg DM/kg cattle LW, on average (Autumn: 8; Winter: 4; Spring: 12, Summer: 8 kg DM/kg LW) and low herbage allowance (LHA) was 5 kg DM/kg cattle LW, on average (Autumn: 4, Winter: 4, Spring: 8 and Summer: 4 kg DM/kg LW). One month before the beginning of the study, forty pregnant heifers Hereford and Aberdeen Angus with an initial mean body weight of 369 ± 5.9 kg and BCS of 4.3 ± 0.08, were randomly assigned to each treatment (20 per treatment), according to breed and body weight. In this trial, three 17-day sampling periods were evaluated: autumn (May 4 to 21, mid gestation), winter (August 4 to 21, late gestation) and spring (November 6 to 23, lactation). The experimental design was a randomized complete generalized block with 2 blocks that represent different soils and two spatial paddocks (replicates) per treatment and per block. Block 1 consisted of Hapludalfs and Argiudolls soils and block 2 of Natruaqolls, Argiudolls and Hapluderts [27]. The area of block 1 was 59 ha and block 2 was 48 ha. The average area per replicate was 13 ± 3.9 ha (4 paddocks/treatment; 5 experimental cows/paddock).

Animal procedures were approved by the Animal Experimentation Committee of Universidad de la República (protocol code 021130-001151-14).

### 2.2. Grazing Management and Herbage Measurements

Before the beginning of the study, experimental cows were managed together and offered the same total herbage allowance in a non-experimental area, with a similar botanical composition as the experimental area. During the trial, experimental animals remained continuously grazing the paddock assigned initially.

HA was adjusted monthly, through the appraisal of available herbage and the “put and take” method [28], using animals of the same physiological status and LW than experimental cows, that were added or removed to adjust animal LW to the intended HA. To estimate herbage mass (kg DM/ha) and sward height (cm), the comparative yield method was used [29], at each measurement date on each paddock. For this purpose, five 0.25 m^2^ quadrants were used as reference to build a herbage mass scale (standards 1–5, lowest to highest yield, respectively). The quadrat samples were collected in triplicate, cut at ground level and dried at 60 °C to express the scale on DM basis. This scale was used to determine regression equations to estimate sward herbage mass on the basis of the assigned score. At the same time, sward height of each quadrant was measured with a stick graduated in centimeters, at the maximum concentration of forage [30]. In each paddock, the herbage mass and sward height were estimated on the basis of a minimum of 100 visual scores generated by three trained observers.

For chemical analysis determination, a composite sample of the herbage was used. The amount of sample material used in each composite was proportional to the frequency of the corresponding standard point of the scale in each paddock.

### 2.3. Animal Measurements

Once a month, experimental cows were weighted and BCS was determined, always by the same experienced evaluator, using the 1–8 scale, [31]. Cow LWs were determined in the morning without fasting [32], and then it was adjusted for uterine weight according to days of gestation [33] based on calving date and assuming 280 days of gestation for all cows. Calving date was September 14 ± 15 days, on average, and they stayed with their mothers for six months until weaning.

Individual herbage organic matter (OM) intake was determined using chromic oxide (Cr_2_O_3_) to estimate fecal OM output, and nitrogen (Nf) and acid detergent fiber (ADFf) contents in feces (g/kg OM) to estimate OM digestibility (OMD) of ingested herbage, according to the equation established by Comeron and Peyraud [34] for herbage-based diets (OMD = 0.791 + 0.0334 Nf − 0.0038 FDAf). Animals were dosed once a day with 10 g of Cr_2_O_3_ from day 1 to 12 of each period of measures. First seven days with the aim of reaching a state of equilibrium at the ruminal level (stabilization period of the marker). From day 8 to 12, feces were rectal-sampled and individual samples were oven dried at 60 °C during 72 h in order to determine the DM content, the Cr_2_O_3_ concentration, and the chemical composition.

The enteric methane emission was measured using the sulfur hexafluoride (SF_6_) tracer gas technique reported by Johnson & Johnson [35] and adapted by Gere and Gratton [36] for a 5-day collection period. Methane sampling equipment and procedures were as reported by Dini et al. [37]. Prior to the start of the first period (15 days earlier), a permeation tube of SF_6_ (with an average daily rate of 3.53 ± 0.271 mg/d) was introduced per os into the rumen of each animal. The breath gas samples were measured over five days in each period (days 13 to 17). The breath gas sampling system consisted of two 0.5 L stainless steel collecting vessels per cow, with a ball-bearing inflow restrictor adjusted to accumulate 0.5 bar of air sample during a 5-day period and a short tube used to connect both. Both inflow restrictors were placed above the animal’s nostrils and protected against water and dust. The two collecting vessels were fitted to each animal’s head by means of especially designed halters. The equipment enabled us to obtain two measurements of methane emission per cow and per period. Immediately prior to the sampling period, each collecting vessel was evacuated (<0.5 mb) after cleaning with high purity nitrogen gas (N_2_). Additionally, an identical set as used with the cows was used to collect background air samples during each 5-day period. The breath gas samples collected were analyzed immediately after the end of the experimental period.

### 2.4. Chemical Analysis

All chemical analyses were conducted at the Laboratory of Animal Nutrition, College of Agronomy, University of the Republic, Uruguay (UDELAR). All the dried samples were ground through a 1 mm screen before chemical analysis. The DM concentration was determined by drying at 105 °C in an oven for 24 h and ash content was determined by incineration at 600 °C for 4 h for organic matter (OM) calculation. The total nitrogen was assayed using the Kjeldahl method (Method 984.13) [38] and expressed as crude protein (CP, nitrogen x 6.25). Content of neutral detergent fiber (NDFom) and acid detergent fiber (ADFom) were determined as described by Van Soest et al. [39], except that the samples were weighted into filter bags and treated with neutral detergent solution that included heat-stable amylase, in ANKOM equipment (ANKOM Technology, Macedon, NY, USA), and expressed as ash-free residues. Gross Energy (GE) was determined using an adiabatic bomb calorimeter (Gallenkamp Autobomb; Loughborough, Leics, UK). Chromium (Cr) concentration in fecal samples was determined by atomic absorption spectrophotometry (Perkin-Elmer 2380, Norwald, CT, USA), using air and an acetylene flame according to William et al. [40]. Chromium standards were combined with fecal samples taken before the experiment began. CH_4_ and SF_6_ concentrations were determined by gas chromatography on an AGILENT 7890 chromatograph. The samples were injected at once in two different setups. For CH_4_, a 3 mL loop, a HP-PLOT Q column and an FID detector were used. For SF_6_, a 10 mL loop, a HP-MOLSIV column, and an ECD detector were used. Each sample was analyzed at least twice, and the average values were used to obtain methane concentration and methane emission. After having chromatographic analyses of samples, CH_4_ emission per animal was calculated using the permeation rate of SF_6_ tube and results obtained from concentration of CH_4_ and SF_6_ [35].

### 2.5. Statistical Analyses

Data were analyzed according to a complete randomized block design using the SAS System program [41], with 2 blocks representing different soil types. Sward characteristics and herbage chemical composition were analyzed using the MIXED procedure with a mixed model that included HA treatment, period, block and their interactions as fixed effects and paddocks as a random effect. Covariance structure used for the repeated measures analysis was Spatial Power Law (SP (POW)) model.

Herbage intake, body weight, BCS and methane emissions were analyzed as repeated measures using the MIXED procedure with the unrestricted covariance structure (UN) and the Kenward–Rogers procedure to adjust the denominator degrees of freedom. The model included HA treatment, period, block and their interactions as fixed effects, and paddock and cow as random effects.

Least square means were compared using the Tukey–Kramer test, and differences were considered to be statistically significant at *p* < 0.05. Data are presented as least square means ± standard errors, in three seasons (autumn, winter and spring).

## 3. Results

### 3.1. Weather Parameters

**Figure 1:**
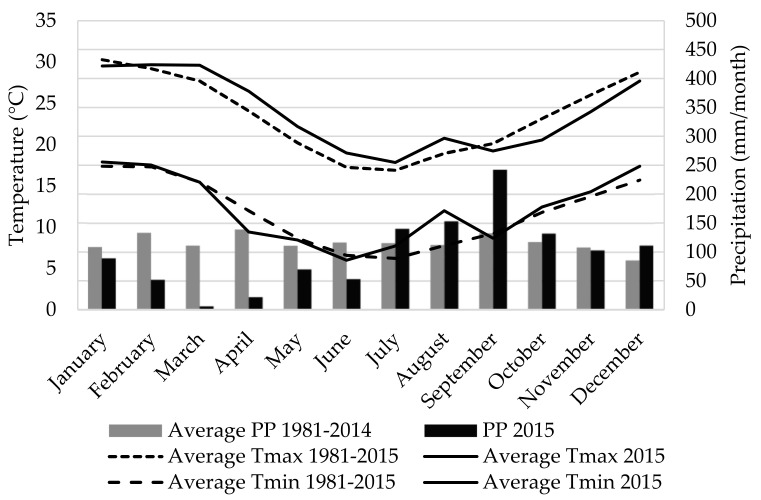
Monthly rainfall (PP) and maximum (Tmax) and minimum (Tmin) daily temperature during 2015 (solid lines) at the experimental site (Cerro Largo, Uruguay), compared to historical average (1981–2015; dashed lines) [24].

Total precipitation was below normal (−16%) throughout the experimental period (2015). In particular, in late summer/early autumn, rainfall accumulation was 50% less than the historical average (1981–2014) for the region. Temperatures were similar to the 30-year mean and greater than the long-term mean only in August 2015 (Figure 1).

### 3.2. Herbage Mass, Height and Chemical Composition

There was an interaction between HA and season (*p* = 0.003, Table 1). In autumn and winter there was no difference between HA treatments for herbage mass (1731 and 1022 kg DM/ha on average), and sward height (5.8 and 3.8 cm on average). However, for spring, the HHA had higher herbage mass (30%) and height (41%) than the LHA. Accordingly, both herbage biomass and sward height differed between treatments, associated with greater accumulation of biomass during late spring (Table 1).

Chemical composition of the offered pasture during the three measurement periods (Table 1), was neither affected by treatments nor between periods, or their interaction, except for the crude protein percentage (CP), which was greater and similar between treatments in late spring (10%).

### 3.3. Digestibility and Intake

There was a significant interaction among OMD and season (*p* = 0.001, Table 2). Herbage allowance did not affect OMD of the selected herbage during autumn and winter, but during spring HHA had higher OMD than LHA. As a result, it was similar between treatments (67.4% on average), but it differed among periods, showing a slightly lower value in autumn (67.1% vs. 67.5% on winter and spring) (Table 2).

Organic matter intake (OMI) estimated from fecal output and digestibility of the selected herbage was similar between treatments, but it differed among periods (6.2 kg OM/d on autumn and winter on average, and 10.8 kg OM/d on spring, *p* < 0.001) (Table 2).

On DM-basis, there was an interaction between treatments and season (*p* = 0.06), with higher dry matter intake (DMI) in HHA (*p* = 0.05) during autumn (8.9 vs. 7.3 kg DM/d), and spring (12.9 vs. 11.1 kg DM/d), while in winter there were no differences.

Both LW and BCS were similar between treatments (340 kg PV and 3.7 BCS, on average), but they differed among periods (*p* < 0.001). The lowest LW and BCS values were reached in August (winter) (288 ± 9.8 kg and 3.3 ± 0.13), but in November (spring) a recovery was observed (383 ± 9.6 kg and 3.8 ± 0.13) (Table 2).

### 3.4. Methane Emission

Daily methane emissions did not differ between treatments, but there was an effect of the measurement period, as spring values were higher than both autumn and winter values. Conversely, percentage of gross energy in feed converted to methane (Ym) was similar among treatments and between periods (7.4% on average). Methane yield as g CH_4_/kg DMI (MY), was also similar between treatments, but it differed among periods (21.7, 22.6 and 26.5 g methane/kg DMI, on autumn, winter, and spring, respectively) (Table 3).

## 4. Discussion

As expected, herbage mass was on average greater in HHA (2409 vs. 1778 ± 356 kg DM/ha in HHA and LHA, respectively, *p* < 0.001). However, herbage accumulation only differed between treatments in the spring. Variable weather conditions at the beginning of the study, might have contributed to the lack of differences observed on herbage accumulation in early autumn among treatments (1731 kg DM/ha on average in autumn), despite the higher herbage allowance assigned on HHA treatment (Table 1). As mentioned previously, at the beginning of the year 2015, in autumn, rainfall was below the average historical records (Figure 1). Herbage accumulation on HHA treatment in autumn might have been restricted by precipitation deficit, that was not override in winter because of decreasing temperatures and photoperiod (1022 kg DM/ha on average in winter for both treatments). During the spring season, when day length and temperatures were favorable, herbage accumulation attained more than 3-fold increase compared to the winter period on both treatments, associated to a better water balance in soil but also, associated to the botanical sward characteristics of Campos grassland (predominance of C4 species) [42]. The different grazing management imposed in HHA resulted in a 30% higher biomass accumulation compared to LHA treatment. Sward height followed the same trend as herbage biomass and reached 40% more height in HHA than in LHA treatment (Table 1). Herbage allowance did not affect herbage chemical composition, as reported previously by Do Carmo et al. [19], for the same grasslands (Table 1). Herbage NDF (71%) and ADF (37%) contents remained high all over the three periods of measures, but CP content differed among periods, being greater in spring than in the other two seasons (10 vs. 7% on average, in spring and autumn–winter, respectively) associated to the regrowth stage of the summer grasses.

DMI value, which varies with the ash content of the sample herbage analyzed, differed between treatments, being higher in HHA in both autumn and spring, and with no differences in winter (Table 2). However, herbage intake expressed as OMI (that is the best estimator of energy intake) did not differ between HHA and LHA treatments in any of the experimental periods, with an average of 7.7 kg OM/d. OMI according to animal characteristics and herbage biomass per unit area in each season, were in agreement with requirements calculated from the standards given by NRC [43]. On the other hand, OMI was higher in spring (10.8 kg OM/d) than the other previous seasons (6.2 kg OM/d autumn–winter on average, *p* < 0.01) (Table 2). Considering sward characteristics to improve intake at grazing, several authors [44,45,46,47,48] reported that herbage biomass per unit area between 1400 and 2500 kg DM/ha, and sward height between 7.5 and 13.5 cm, were considered optimal to maximize cattle intake in native grasslands of Rio Grande do Sul (southern Brazil). In this experiment, herbage biomass in autumn was close to reference values reported by these authors (1731 kg DM/ha on average), but sward height remained lower (5.8 cm on average, Table 1).

On the other hand, grazing management adjusting seasonally herbage allowance, did have an effect on herbage mass accumulation and sward height in spring, but OMI remained similar between treatments. In this season, we can assume that the herbage allowance assigned to treatments (12 and 8 kg DM/kg LW) allowed cows to graze nearly of their potential intake. Concerning selectivity, the herbage OM digestibility of herbage consumed in spring differed among treatments (68.0% vs. 67.3% for HHA and LHA, respectively, Table 2), but the difference was not reflected in better live weight or BCS for cows grazing HHA.

Enteric methane emissions values did not differ among treatments as neither the quantity nor the quality of the herbage consumed by cows varied according to HA treatments. However, season did have an effect associated with the increase in herbage intake in spring (159, 150, 312 g CH_4_/day, on autumn, winter and spring, respectively), doubling the other two periods. As forage consumed increased in spring, total methane emissions were higher [48,49,50], but it must be said that methane measured in spring for LHA was higher than expected considering the amount of OMI in this treatment. Daily methane emissions ranges in this study were similar to prior research in cattle fed grasses [51,52,53,54,55,56,57].

MY (g /kg DMI) averaged 23.6 g/kg DMI among treatments. This average was close to 23.3 g/kg DMI provided by the Intergovernmental Panel on Climate Change (IPCC) [58] for cattle with a diet based on forage and in agreement with previous results reported by international literature for beef cows on native grasslands [59,60,61]. This value of MY was higher than values reported earlier for dairy cows grazing temperate grass-legume mixed pastures in Uruguay [37,62], likely due to the lower forage nutritive value of the native pastures. Kamra et al. [63], suggest that there is a higher methane production associated with feeding with C4 species, probably due to a higher content of non-structural carbohydrates and lignin, lower intake and slower passage rate [64]. Archimède et al. [65] reported that C4 grass fiber tends to be more lignified and more resistant to physical and microbial digestion compared to C3 grasses. So, it seems likely that Campos grassland might produce greater amounts of methane per unit DMI than template pastures, because of warm grasses predominance. Thus, methane as a proportion of the ingested GE (Ym), resulted in 7.4% on average between treatments and seasons (autumn, winter and spring). From our results, Ym ranged within the default Tier 1 value of the IPCC [58] for cattle with more than 75% of forage in the diet (7.0% ± 1.4), but it was smaller than reported values averaging 7.9% in native pastures [50].

Methane emission factor (MEF) calculated using IPCC 2019 [58] (Tier 2 methodology, eqn 10.21a) with data of this study results in 78 kg CH_4_/head/year, similar to values obtained for beef heifers in Canada [66], but higher than the IPCC reference value for methane emission from beef cattle in South America of 56 kg per animal per year. However, this average takes into account the entire beef production system, including fattening and feedlot, so it would be expected to be higher for the cow–calf phase.

On the basis of the datasets available, a single, global MY value or percentage of gross energy in feed converted to methane value, Ym, might not be appropriate for use in Intergovernmental Panel on Climate Change (IPCC) greenhouse accounting methods around the world. Therefore, ideally country specific MEF values should be used in each country’s accounts (i.e., an IPCC Tier 2 or 3 approaches) from data generated within that country [61]. Thus, the results obtained in this study for breeding systems in native grasslands in Uruguay represent the first assessment to a national database on grazing emissions from breeding cows aiming to improve the National GHG Inventory calculations.

## 5. Conclusions

Grazing management adjusted seasonally created fluxes in the production and accumulation of herbage that increased herbage mass in autumn and spring, mainly in HHA treatment. However, herbage intake did not differ among herbage allowance treatments. In autumn, herbage biomass per hectare was close to reference optimal values reported by the literature for Campos grassland, but sward height remained lower and so canopy architecture probably affected herbage intake through their effect on the ease of prehension of herbage. For spring season, we can assume that the herbage allowance assigned allowed cows to graze nearly their potential intake in both treatments, and so with no differences among them.

Since quantity and quality of herbage intake did not differ with herbage allowance, methane yield remained similar between treatments and it was 23.6 g CH_4_/kg DMI or 7.4% on average. These values were higher than those reported earlier, likely due to the lower forage nutritive value of the native pastures. The results obtained in this study for cow–calf systems in native grasslands, during three relevant periods of the productive cycle (medium gestation, final gestation, and lactation), provide unique information to breeding systems in Campos grassland. However, it is crucial to analyze the animal performance in stabilized pastures in terms of biomass per unit area and height according to the herbage allowance evaluated in this work. Furthermore, it is necessary increase the national database on grazing emissions from breeding cows, including more measurements throughout the production cycle in natural grasslands with improved grazing management.

## Figures and Tables

**Table 1:** Mean and standard error of the mean (SEM) of herbage mass, sward height and chemical composition of the offered forage for high (HHA) and low (LHA) herbage allowance treatment and season (S), and their interaction (HA*S) on native grasslands in Cerro Largo, Uruguay grazed by beef cows. Means within the same row followed by different letters are significantly different at *p* < 0.05.

-	Autumn		Winter		Spring			*p*-Value		
	HHA	LHA	HHA	LHA	HHA	LHA	SEM	HA	S	HA*S
Herbage mass (kg DM/ha)	1948 ^bc^	1515 ^cd^	1134 ^cd^	910 ^d^	4147 ^a^	2910 ^b^	355.6	<0.001	<0.001	0.003
Sward height (cm)	6.5 ^b^	5.1 ^bc^	4.1 ^c^	3.4 ^c^	11.6 ^a^	6.8 ^b^	0.77	<0.001	<0.001	<0.001
DM (%) ^1^	91	90	90	90	89	90	1.2	ns	ns	ns
OM (%) ^2^	85	86	86	89	89	90	4.4	ns	ns	ns
CP (%) ^3^	7 ^b^	7 ^b^	7 ^b^	6 ^b^	10 ^a^	10 ^a^	0.3	ns	<0.05	ns
NDF (%) ^4^	78	64	72	74	69	71	4.6	ns	ns	ns
ADF (%) ^5^	43	34	37	36	35	35	2.7	ns	ns	ns
GE (MJ/kg DM) ^6^	17	16	16	18	18	18	0.2	ns	ns	ns

^1^ DM: Dry matter, ^2^ OM: Organic matter, ^3^ CP: Crude protein, ^4^ NDF: Neutral detergent fiber, ^5^ ADF: Acid detergent fiber, ^6^ GE: Gross energy.

**Table 2:** Mean and standard error of the mean (SEM) of fecal output, OMD, OMI and DMI per day, LW and BCS by grazing pregnant heifers on high (HHA) and low (LHA) herbage allowance (HA) treatments in three seasons (S), and their interaction (HA*S), on native grasslands in Cerro Largo, Uruguay. Means within the same row followed by different letters are significantly different at *p* < 0.05.

-	Autumn		Winter		Spring		-	*p*-Value		
	HHA	LHA	HHA	LHA	HHA	LHA	SEM	HA	S	HA*S
Fecal output (kg OM/d)	2.3 ^b^	2.0 ^b^	1.9 ^b^	2.1 ^b^	3.6 ^a^	3.3 ^a^	0.13	0.370	<0.001	0.200
OMD ^1^ (g/kg OM)	670 ^c^	672 ^bc^	673 ^bc^	675 ^ab^	680 ^a^	673 ^bc^	1.9	0.390	0.003	0.001
OMI ^2^ (kg/d)	6.7 ^b^	6.1 ^b^	5.7 ^b^	6.4 ^b^	11.4 ^a^	10.1 ^a^	0.42	0.300	<0.001	0.130
DMI ^3^ (kg/d)	8.9 ^c^	7.3 ^d^	6.6 ^d^	7.2 ^d^	12.9 ^a^	11.1 ^b^	0.46	0.050	<0.001	0.060
LW^4^ (kg)	353 ^b^	345 ^b^	281 ^c^	295 ^c^	380 ^a^	386 ^a^	8.2	0.687	<0.001	0.449
BCS	4.0 ^a^	4.0 ^a^	3.2 ^b^	3.3 ^b^	3.7 ^a^	3.8 ^a^	0.11	0.744	<0.001	0.909

^1^ Organic matter digestibility, ^2^ Organic matter intake, ^3^ Dry matter intake, ^4^ Live weight^,^ adjusted for uterine weight.

**Table 3:** Mean and standard error of the mean (SEM) of daily methane emission and methane yield by grazing pregnant heifers on high (HHA) and low (LHA) herbage allowance (HA) treatments in three seasons (S), and their interaction (HA*S), on native grasslands in Cerro Largo, Uruguay. Means within the same row followed by different letters are significantly different at *p* < 0.05.

-	Autumn		Winter		Spring		-	*p*-Value		
	HHA	LHA	HHA	LHA	HHA	LHA	SEM	HA	S	HA*S
Methane emission (g CH_4_/a/d)	170 ^b^	148 ^b^	140 ^b^	160 ^b^	294 ^a^	329 ^a^	13.3	0.350	<0.001	0.080
Methane yield										
as Gross Energy intake (Ym)	6.3 ^b^	7.2 ^ab^	7.6 ^ab^	7.5 ^ab^	7.2 ^ab^	8.6 ^a^	0.57	0.120	0.140	0.410
as g CH_4_/kg DMI (MY)	21.7 ^b^	21.7 ^b^	21.6 ^b^	23.7 ^ab^	23.9 ^ab^	29.1 ^a^	2.00	0.150	0.040	0.400

## Data Availability

The data presented in this study are available on request from the corresponding author.

## Abbreviations

HA: Herbage allowance, DM: Dry matter, LW: Live weight, MY: Methane yield as g CH4/kg DMI, Ym: Methane yield as a percentage of Gross Energy intake, CH4: Methane, d: day, OM: Organic matter, DMI: Dry matter intake, OMI: Organic matter intake, GHG: Greenhouse gases, HHA: High herbage allowance, LHA: Low herbage allowance, SEM: Standard error of the mean, S: Season.
